# Accuracy of the Radiographic Assessment of Lung Edema Score for the Diagnosis of ARDS

**DOI:** 10.3389/fphys.2021.672823

**Published:** 2021-05-26

**Authors:** Claudio Zimatore, Luigi Pisani, Valeria Lippolis, Melissa A. Warren, Carolyn S. Calfee, Lorraine B. Ware, Anna Geke Algera, Marry R. Smit, Salvatore Grasso, Marcus J. Schultz

**Affiliations:** ^1^Department of Intensive Care, Academic Medical Center, Amsterdam, Netherlands; ^2^Department of Emergency and Organ Transplantation, School of Medicine, University of Bari Aldo Moro, Bari, Italy; ^3^Mahidol-Oxford Tropical Medicine Research Unit (MORU), Mahidol University, Bangkok, Thailand; ^4^Department of Anesthesia and Perioperative Medicine, Regional General Hospital F. Miulli, Acquaviva delle Fonti, Italy; ^5^Department of Intensive Care, Mater Dei Hospital, Bari, Italy; ^6^Division of Allergy, Pulmonary and Critical Care Medicine, Department of Medicine, Vanderbilt University School of Medicine, Nashville, TN, United States; ^7^Department of Medicine and Department of Anesthesia, Cardiovascular Research Institute, University of California, San Francisco, San Francisco, CA, United States; ^8^Department of Pathology, Microbiology and Immunology, Vanderbilt University School of Medicine, Nashville, TN, United States

**Keywords:** invasive ventilation, acute respiratory distress syndrome (ARDS), lung imaging, chest X ray, chest radiogprahs, diagnostic capacity, prognostic capacity, rale score

## Abstract

**Background:** Bilateral opacities on chest radiographs are part of the Berlin Definition for Acute Respiratory Distress Syndrome (ARDS) but have poor interobserver reliability. The “Radiographic Assessment of Lung Edema” (RALE) score was recently proposed for evaluation of the extent and density of alveolar opacities on chest radiographs of ARDS patients. The current study determined the accuracy of the RALE score for the diagnosis and the prognosis of ARDS.

**Methods:** *Post-hoc* analysis of a cohort of invasively ventilated intensive care unit (ICU) patients expected to need invasive ventilation for >24 h. The Berlin Definition was used as the gold standard. The RALE score was calculated for the first available chest radiograph after start of ventilation in the ICU. The primary endpoint was the diagnostic accuracy for ARDS of the RALE score. Secondary endpoints included the prognostic value of the RALE score for ICU and hospital mortality, and the association with ARDS severity, and the PaO_2_/FiO_2_. Receiver operating characteristic (ROC) curves were constructed, and the optimal cutoff was used to determine sensitivity, specificity and the negative and positive predictive value of the RALE score for ARDS.

**Results:** The study included 131 patients, of whom 30 had ARDS (11 mild, 15 moderate, and 4 severe ARDS). The first available chest radiograph was obtained median 0 [0 to 1] days after start of invasive ventilation in ICU. Compared to patients without ARDS, a higher RALE score was found in patients with ARDS (24 [interquartile range (IQR) 16–30] *vs.* 6 [IQR 3–11]; *P* < 0.001), with RALE scores of 20 [IQR 14–24], 26 [IQR 16–32], and 32 [IQR 19–36] for mild, moderate and severe ARDS, respectively, (*P* = 0.166). The area under the ROC for ARDS was excellent (0.91 [0.86–0.96]). The best cutoff for ARDS diagnosis was 10 with 100% sensitivity, 71% specificity, 51% positive predictive value and 100% negative predictive value. The RALE score was not associated with ICU or hospital mortality, and weakly correlated with the PaO_2_/FiO_2_.

**Conclusion:** In this cohort of invasively ventilated ICU patients, the RALE score had excellent diagnostic accuracy for ARDS.

## Introduction

The chest radiograph is a frequently used imaging tool in intensive care unit (ICU) patients (Trotman-Dickenson, 2003; Graat et al., 2006), although its clinical value has been disputed (Graat et al., 2005). Findings on chest radiographs are an important part of the Berlin Definition for acute respiratory distress syndrome (ARDS Definition Task Force et al., 2012), despite the low interobserver reliability that does not improve with training (Rubenfeld et al., 1999; Goddard et al., 2018). Also, the description of chest radiographs findings remains mostly subjective. Recently, therefore, the “Radiographic Assessment of Lung Edema” (RALE) score was proposed (Warren et al., 2018), a numeric scoring system in which the chest is divided into four quadrants that are each scored on a numerical scale for extent of consolidation *and* density of opacification. The RALE score is calculated by summing the product of the scores for consolidation and density of opacification of the four quadrants, and can range from 0 to 48.

While the first description of the RALE score focused on validating the score against gravimetric quantification and testing the association between the score and outcome in patients with ARDS (Warren et al., 2018), it could be that this score also has discriminating properties to diagnose ARDS in invasively ventilated ICU patients who may or may not have ARDS. In addition, with every new scale or score, it is necessary to externally validate its capacity, feasibility and reliability (Patrick and Chiang, 2000; Keszei et al., 2010; Kottner et al., 2011).

The objective of the current study was two–fold. The first objective was to determine whether the RALE score has diagnostic properties for ARDS, and prognostic properties in ICU patients. The second objective was to assess the feasibility and interobserver reliability of the RALE score. These objectives were studied using the chest radiographs of patients in a well–defined cohort of invasively ventilated ICU patients (Vercesi et al., 2018). The hypotheses tested were that the RALE score has a good diagnostic accuracy for ARDS, and that the RALE score has prognostic value in invasively ventilated ICU patients, independent of the diagnosis of ARDS.

## Materials and Methods

### Study Design and Settings

This study was a *post-hoc* analysis of a single–center observational study performed in the ICU of the Amsterdam University Medical Centers, location Academic Medical Center (AMC) between November 2016 and June 2017 (Vercesi et al., 2018; Pisani et al., 2019). The Institutional Review Board of the AMC approved the original study and waived the need for informed consent from individual patients because data used in this study had been collected as part of standard care for patients with acute respiratory failure (approval W17_353 # 17.411).

### Inclusion and Exclusion Criteria

Patients were eligible for participation in the original study if they: (a) were expected to receive invasive ventilation for at least 24 h at the moment of screening, (b) received ventilation with a minimum of 5 cm H_2_O positive end–expiratory pressure (PEEP); and (c) had a chest radiograph or lung CT scan within the first 24 and 48 h of start of invasive ventilation, respectively. As the original study focused on the diagnostic value of lung ultrasound plus pulse oximetry for moderate or severe ARDS, the original study had two exclusion criteria, namely: (a) no lung ultrasound study made within 48 h of start of invasive ventilation; and (b) conditions potentially compromising reliability of pulse oximetry, including carbon monoxide poisoning. The number of excluded patients because of these reasons, though, was very low. An additional exclusion criterion for the current analysis was the absence of a chest radiograph during the first 2 days of invasive ventilation in the ICU.

### Data Collection

Collection of data involved demographic characteristics including age, gender, height, weight, and body mass index; disease severity scores including the acute physiology and chronic health evaluation IV score and the simplified acute physiology score II; and ventilation characteristics including FiO_2_, minute volume, PEEP, maximum airway pressure (P_max_), respiratory rate, tidal volume, and blood gas analysis results.

### ARDS Diagnosis

Acute respiratory distress syndrome was diagnosed according to the Berlin Definition for ARDS (ARDS Definition Task Force et al., 2012). For this, a panel of independent experienced clinicians assessed presence or absence of ARDS, strictly using the 4 components of the Berlin Definition for ARDS, i.e., new or worsening respiratory symptoms within 1 week of a known medical clinical insult; a PaO_2_/FiO_2_ < 300 mm Hg at a minimum of 5 cm H_2_O PEEP; bilateral opacities on the chest radiograph or computed tomography (CT) exam, not explained by effusions, collapse or nodules; and respiratory failure not fully explained by cardiac failure or fluid overload. Of note, the clinicians applying the criteria in the Berlin Definition for ARDS could not calculate the RALE score, as this score was developed and reported in the literature after their assessments.

### RALE Score

**FIGURE 1:**
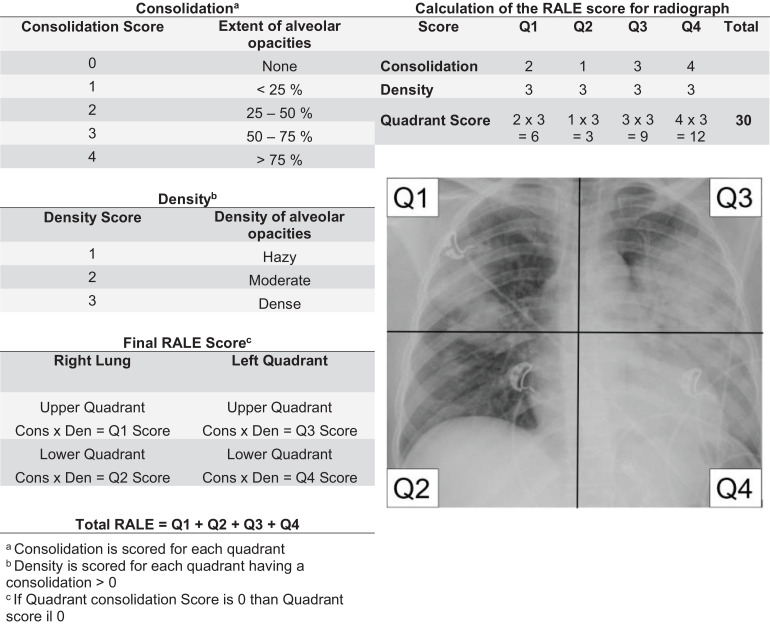
Consolidation and density scoring in the RALE score.

Two independent researchers (CZ and VL) scored the first available chest radiograph after start of mechanical ventilation in ICU patients. These researchers were unaware of clinical information or presence or absence of ARDS, as well as the results of assessments of the above–mentioned physicians who applied the criteria in the Berlin Definition. In short, as shown in Figure 1, the lung fields on the chest radiograph were divided into four quadrants by a vertical line over the spine and a horizontal line at the level of the first branch of the left main bronchus, exactly as described in the seminal publication on the RALE score (Warren et al., 2018). Each quadrant was assigned a number, and the extent of alveolar opacities (the consolidation score, from 0 to 4) and density of alveolar opacities (the density score, from 1 to 3) was determined. If the consolidation score was 0, the density score was 0. The final RALE score was the sum of the product of the consolidation and density score for each quadrant. Thus, the final RALE score ranged from minimum 0 to maximum 48.

### Endpoints

The primary endpoint was the diagnostic accuracy for ARDS of the RALE score. Secondary endpoints included the prognostic value of the RALE score for ICU and hospital mortality, correlation between the RALE score and ARDS severity, and the inter-observer reliability for the RALE scoring, the correlation with the PaO_2_/FiO_2_ at the moment the chest radiograph was obtained.

### Statistical Analysis

Demographic data, and clinical and outcome variables were presented as frequencies with percentages for categorical variables and as medians with interquartile ranges for continuous variables.

To determine the reliability of the RALE score, the interobserver variability (Keszei et al., 2010) between the primary scorer and a second independent investigator was tested on the entire cohort of the patients. For this, a two–way mixed consistency average measures intraclass correlation coefficient (ICC) was calculated. A Bland–Altman plot and a scatter plot were used to visualize the agreement between independent viewers. For the primary analysis only the scores attributed by the primary scorer were used.

To determine the diagnostic accuracy of the RALE score for ARDS, the Area Under the Receiver Operating Characteristic curve (AUROC) with 95% confidence intervals (CI) was calculated. Diagnostic accuracy was considered “excellent” if AUROC was between 0.9 and 1, “very good” between 0.8 and 0.9, “good” between 0.7 and 0.8, “sufficient” between 0.6 and 0.7, and “bad” between 0.5 and 0.6 (Šimundić, 2009). The best cutoffs, the maximum difference between true positive and false positive, were obtained with the Youden index (Youden, 1950) (sensitivity + specificity – 1). Sensitivity, specificity, positive and negative predictive values were calculated using this cutoff.

Next, RALE scores were compared between patients without ARDS, and patients with mild, moderate or severe ARDS, and local polynomial regression (LOWESS curve fitting) was used to assess the correlation between RALE score with PaO_2_/FiO_2_, PEEP, FiO_2_, and P_Max_.

Finally, to determine the prognostic accuracy for ICU or hospital mortality, ROCs were constructed and analyzed in the same way as for determining the diagnostic accuracy for ARDS.

Statistical significance was considered when *P* < 0.05. All analyses were performed using IBM SPSS Statistics 24.0 and graphs built using Prism 8 (GraphPad software, version 8.4.2).

## Results

### Patients

**FIGURE 2:**
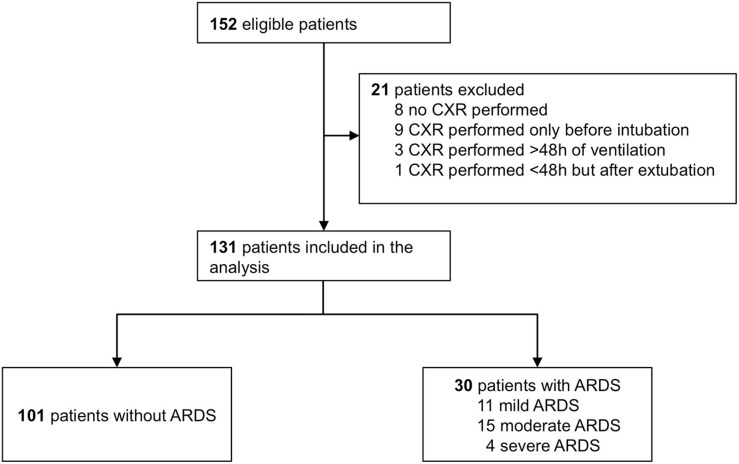
Patient flow.

Patient flow is shown in Figure 2. Of the 152 patients in the original cohort, 131 patients fulfilled the additional criteria for participation in the current analysis. Of them, 101 were diagnosed as not having ARDS, and 30 fulfilled the Berlin Definition for ARDS (11, 15, and 4 patients with mild, moderate and severe ARDS, respectively). Demographic and ventilatory characteristics are presented in Table 1.

**TABLE 1:** Baseline characteristics, outcomes and ventilatory characteristics of patients at the moment of the chest radiograph.

	**No ARDS**	**Mild ARDS**	**Moderate ARDS**	**Severe ARDS**
**Baseline characteristics and outcomes**				
Age, years	62 [51,72]	65 [52,71]	55 [24,69]	54 [37,59]
Gender, male	59 (58)	5 (45)	11 (73)	3 (75)
BMI, kg/m^2^	25 [23,28]	26 [24,28]	26 [23,33]	24 [23,29]
SAPS	37 [15,52]	24 [15,53]	20 [3,49]	52 [12,61]
SOFA	10 [7,12]	9 [6,11]	11 [8,13]	13 [11,15]
APACHE II score	22 [17,27]	20 [15,31]	19 [13,24]	24 [15,27]
ICU mortality	32 (32)	3 (27)	3 (20)	4 (100)
Hospital mortality	41 (41)	3 (27)	3 (20)	4 (100)
Duration of ventilation	3 [1,8]	5 [2,17]	12 [7,18]	11 [1,21]
VFD 28, days	19 [0,25]	18 [0,24]	15 [0,20]	0 [0,0]
**Ventilation characteristics***x*				
FiO_2_, %	40 [30,50]	50 [40,65]	50 [40,60]	100 [93,100]
Minute volume, L/min	8.7 [6.9,10.8]	10.3[9.3,12.9]	10.8[9.4,12.7]	11.4[4.9,13.5]
PEEP, cm H_2_O	5 [5,8]	8 [7,10]	12 [8,15]	15 [11,15]
P_*max*_, cm H_2_O	22 [16,27]	28 [26,35]	31 [27,36]	35 [31,39]
RR measured, breath/min	18 [16,24]	26 [22,32]	27 [21,30]	23 [13,30]
V_*T*_, ml/kg PWB	8.2 [6.7,9.3]	8.5 [6.1,10.1]	6.9 [4.8,8.8]	5.4 [2.6,9.4]
SpO_2_, %	98 [96,100]	96 [93,99]	95 [94,98]	89 [78,95]
PaO_2_, mm Hg	89 [80,102]	80 [69,93]	77 [67,80]	75 [61,92]
PaO2 to FiO2 ratio	253 [173,321]	159 [131,212]	143 [123,177]	78 [62,92]

*Values are medians [interquartile range] or numbers (percentage). Abbreviations: ARDS, acute respiratory distress syndrome; SAPS, simplified acute physiology score; SOFA, sequential organ failure assessment; APACHE, acute physiology and chronic health; VFD 28, ventilator-free days at day 28; FiO2, fraction of inspired oxygen; PEEP, positive end–expiratory pressure; Pmax, maximum airway pressure; RR, respiratory rate; VT, tidal volume; PBW, predicted body weight; SpO2, pulse oximetry saturation; PaO2, arterial oxygen pressure; and PaCO2, arterial carbon dioxide pressure.*

The ICC for applying the RALE score was excellent (0.95 [95%– CI 0.92–0.96]). The Bland–Altman plot showed a strong agreement and the scatter plot suggests high degree of agreement between the two independent researchers (Supplementary Figure 1).

### The Diagnostic Performance of the RALE Score

**FIGURE 3:**
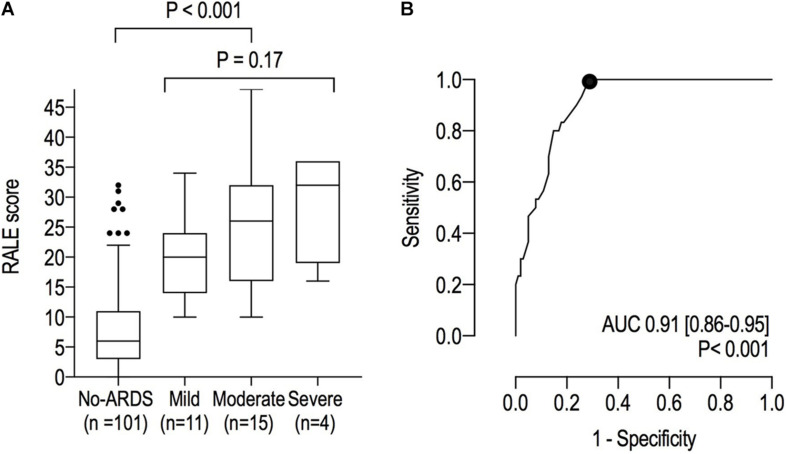
**(A)** RALE scores in patients with ARDS and those without ARDS; **(B)** Receiver Operating Characteristic curves for ARDS. The dot represents the optimal cutoff.

The RALE score was higher in ARDS patients compared to patients without ARDS (24 [16–30] *vs.* 6 [3–11]; *P* < 0.001) and had an excellent area under the ROC for ARDS (Figure 3). The best cutoff for ARDS diagnosis was 10 (Youden’s index = 0.710) with 100% sensitivity, 71% specificity, 51% positive predicted value and 100% negative predicted value. Although the RALE scores increased with ARDS severity, differences between the severity groups were non–significant (20 [14–24], 26 [16–32] and 32 [19–36] in mild, moderate and severe ARDS, respectively; *P* = 0.166).

### The Prognostic Value of the RALE Score

**FIGURE 4:**
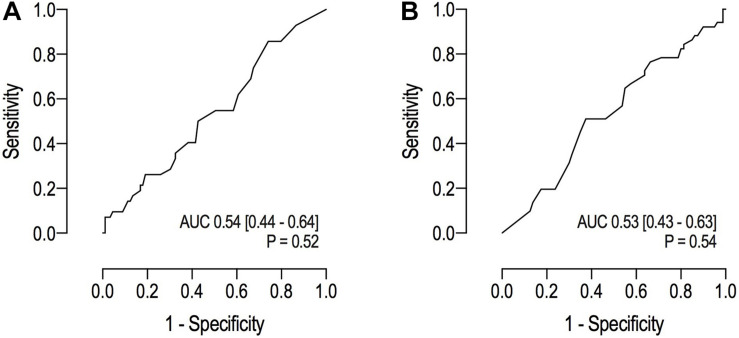
**(A)** Receiver operating characteristic curves for ICU mortality; **(B)** Receiver operating characteristic curves for hospital mortality.

The prognostic capacity of the RALE score for ICU – and hospital mortality was poor (Figure 4).

### Correlation With PaO_2_/FiO_2_

The correlation between RALE score and PaO_2_/FiO_2_ was weak (*R*^2^ linear = 0.21; Supplementary Figure 2). No meaningful association was detectable between the RALE score and PEEP levels recorded at the moment of the CXR.

## Discussion

The findings of this *post-hoc* analysis of cohort of well–defined invasively ventilated critically ill patients expected not to be extubated within 24 h can best be summarized as follows: (a) the RALE score is higher in patients with ARDS compared to patients not fulfilling the Berlin Definition for ARDS, (b) the diagnostic performance for ARDS of the RALE score is excellent, with a cutoff of 10 showing excellent sensitivity and moderate specificity; (c) though has poor prognostic value in a mixed cohort of patients with may or may not have ARDS; (d) the RALE score increases from mild to severe ARDS, though this finding was not statistically significant; and (e) the RALE score correlates weakly with the PaO_2_/FiO_2_.

This study has several strengths. It used the data of a prospective study in which consecutive patients expected to be intubated for at least 24 h were included. The original study as well as the current re–analysis had only few exclusion criteria, increasing its generalizability. Only eight patients were excluded because of a missing chest radiograph. The chest radiographs used for calculating the RALE score were as close as possible to start of invasive ventilation in the ICU, and always with a PEEP ≥ 5 cm H_2_O. ARDS was diagnosed using the present “gold standard,” i.e., the Berlin Definition for ARDS, applied by independent physicians with extensive experience in using it. Finally, as a measure against bias, clinicians involved in applying the criteria in the Berlin Definition for ARDS were unaware of the RALE score, and *vice versa*, the investigators calculating the RALE score remained blinded for the presence of absence of ARDS.

One salient finding was the high agreement between the two researchers with regard to the RALE score in individual cases. This new numeric score seemed easy to learn and calculate, and gave a uniform interpretation of chest radiographs, in line with the seminal report on use of the RALE score (Warren et al., 2018). It is noticeable that the Berlin Definition investigators demonstrated low interobserver reliability which did not improve with training (Goddard et al., 2018). Thus, one could argue to use this new score as a finding to make diagnosing ARDS easier.

The findings of the current study are at least in part in line with the finding in the seminal study on this new score, i.e., that higher RALE scores are found in patients with more injured lungs, according to the PaO_2_/FiO_2_. One difference between the two studies was that in the current study the RALE score was calculated in much “broader” population of invasively ventilated ICU patients, i.e., not only patients with ARDS, but also patients at risk of this complication. The RALE score demonstrated an excellent diagnostic accuracy for ARDS, and may be taken into consideration in future refinements of the radiological criteria of the Berlin Definition of ARDS. The increase in RALE score from mild, to moderate and severe ARDS was not statistically significant, in agreement with a recent study focusing on the evolution of the RALE score in 108 patients with ARDS (Kotok et al., 2020). However, it must be mentioned that the number of patients with ARDS, in particular severe ARDS, was low.

Although we could not find an association between baseline RALE and mortality, a recent study proposes that the change in RALE score in the first days is associated with survival in ARDS (Jabaudon et al., 2020). Also in patients with pneumonia from coronavirus disease, both the visually scored and RALE score and the ones computed from artificial intelligence algorithms were associated with poor outcomes (Ebrahimian et al., 2021).

While the RALE score had a weak association with ARDS categories based on degree of hypoxemia, scores could independently increase the diagnostic performance and the outcome prediction. This should be tested in future cohorts of invasively ventilated ICU patients. This study has other limitations. The study included a relatively small number of patients, resulting in a low number of patients with ARDS, and especially few patients with severe ARDS. In addition, this was a single center study with all available patients being used without a formal power calculation performed beforehand. It will be important to confirm the results of the current study performing the RALE score in a multicenter setting.

In conclusion, the RALE score provides a reliable interpretation of signs of lung edema on chest radiographs in invasively ventilated ICU patients. The RALE score has an excellent diagnostic accuracy for ARDS in such patients but has only a weak correlation with PaO_2_/FiO_2_ and no associations with patient outcomes. Additional validation of the cutoff and performance of the RALE score is needed in larger cohorts.

## Data Availability Statement

The raw data supporting the conclusions of this article will be made available by the authors, without undue reservation.

## Ethics Statement

Ethical review and approval was not required for the study on human participants in accordance with the local legislation and institutional requirements. Written informed consent for participation was not required for this study in accordance with the national legislation and the institutional requirements.

## Author Contributions

MJS, LW, CC, MW, SG, CZ, and LP contributed to conception and design of the study. CZ, LP, and VL organized the database. CZ and LP performed the statistical analysis. CZ wrote the first draft of the manuscript. LP, VL, AA, and MRS wrote sections of the manuscript. MJS and LP supervised the project and revised the present manuscript. All authors contributed to the manuscript revision, read, and approved the submitted version.

## Conflict of Interest

The authors declare that the research was conducted in the absence of any commercial or financial relationships that could be construed as a potential conflict of interest.

## References

[B1] ARDS Definition Task Force Ranieri V. M. Rubenfeld G. D. Thompson B. T. Ferguson N. D. Caldwell E. (2012). Acute respiratory distress syndrome. *JAMA.* 307 2526–2533. 10.1001/jama.2012.5669 22797452

[B2] Ebrahimian S. Homayounieh F. Rockenbach M. A. B. C. Putha P. Raj T. Dayan I. (2021). Artificial intelligence matches subjective severity assessment of pneumonia for prediction of patient outcome and need for mechanical ventilation: a cohort study. *Sci. Rep.* 11:858. 10.1038/s41598-020-79470-0 33441578 PMC7807029

[B3] Goddard S. L. Rubenfeld G. D. Manoharan V. Dev S. P. Laffey J. Bellani G. (2018). The randomized educational acute respiratory distress syndrome diagnosis study: a trial to improve the radiographic diagnosis of acute respiratory distress syndrome. *Crit. Care Med.* 46 743–748. 10.1097/ccm.0000000000003000 29438110

[B4] Graat M. E. Hendrikse K. A. Spronk P. E. Korevaar J. C. Stoker J. Schultz M. J. (2006). Chest radiography practice in critically ill patients: a postal survey in the Netherlands. *BMC Med. Imaging* 6:8. 10.1186/1471-2342-6-8 16848892 PMC1557847

[B5] Graat M. E. Stoker J. Vroom M. B. Schultz M. J. (2005). Can we abandon daily routine chest radiography in intensive care patients? *J. Intensive Care Med.* 20 238–246. 10.1177/0885066605277212 16061907

[B6] Jabaudon M. Audard J. Pereira B. Jaber S. Lefrant J. Y. Blondonnet R. (2020). Early changes over time in the radiographic assessment of lung edema score are associated with survival in ARDS. *Chest* 158:2394. 32659235 10.1016/j.chest.2020.06.070 PMC7768934

[B7] Keszei A. P. Novak M. Streiner D. L. (2010). Introduction to health measurement scales. *J. Psychosom. Res.* 68 319–323. 10.1016/j.jpsychores.2010.01.006 20307697

[B8] Kotok D. Yang L. Evankovich J. W. Bain W. Dunlap D. G. Shah F. (2020). The evolution of radiographic edema in ARDS and its association with clinical outcomes: a prospective cohort study in adult patients. *J. Crit. Care* 56 222–228. 10.1016/j.jcrc.2020.01.012 32028223 PMC7136845

[B9] Kottner J. Audige L. Brorson S. Donner A. Gajewski B. J. Hróbjartsson A. (2011). Guidelines for reporting reliability and agreement studies (GRRAS) were proposed. *Int. J. Nurs. Stud.* 48 661–671. 10.1016/j.ijnurstu.2011.01.016 21514934

[B10] Patrick D. L. Chiang Y. P. (2000). Measurement of health outcomes in treatment effectiveness evaluations: conceptual and methodological challenges. *Med. Care.* 38(suppl. 9)II, 14–25. 10.1097/00005650-200009002-00005 10982087

[B11] Pisani L. Vercesi V. van Tongeren P. S. I. Lagrand W. K. Leopold S. J. Huson M. A. M. (2019). The diagnostic accuracy for ARDS of global versus regional lung ultrasound scores - a post hoc analysis of an observational study in invasively ventilated ICU patients. *Intensive Care Med. Exp.* 7(Suppl. 1):44. 10.1186/s40635-019-0241-6 31346914 PMC6658630

[B12] Rubenfeld G. D. Caldwell E. Granton J. Hudson L. D. Matthay M. A. (1999). Interobserver variability in applying a radiographic definition for ARDS. *Chest* 116 1347–1353. 10.1378/chest.116.5.1347 10559098

[B13] Šimundić A.-M. (2009). Measures of diagnostic accuracy: basic definitions. *EJIFCC* 19 203–211. 27683318 PMC4975285

[B14] Trotman-Dickenson B. (2003). Radiology in the intensive care unit (Part I). *J. Int. Care Med.* 18 198–210. 10.1177/0885066603251897 15035766

[B15] Vercesi V. Pisani L. van Tongeren P. S. I. Lagrand W. K. Leopold S. J. Huson M. M. A. (2018). External confirmation and exploration of the kigali modification for diagnosing moderate or severe ARDS. *Intensive Care Med.* 44 523–524. 10.1007/s00134-018-5048-5 29368056

[B16] Warren M. A. Zhao Z. Koyama T. Bastarache J. A. Shaver C. M. Semler M. W. (2018). Severity scoring of lung oedema on the chest radiograph is associated with clinical outcomes in ARDS. *Thorax* 73 840–846. 10.1136/thoraxjnl-2017-211280 29903755 PMC6410734

[B17] Youden W. J. (1950). Index for rating diagnostic tests. *Cancer* 3 32–35. 10.1002/1097-0142(1950)3:1<32::aid-cncr2820030106>3.0.co;2-3 15405679

