# Supplementary material for: Accuracy of the Radiographic Assessment of Lung Edema Score for the Diagnosis of ARDS
Source: Front Physiol. 2021 May 26;12:672823. doi: 10.3389/fphys.2021.672823 (PMC8188799; doi:10.3389/fphys.2021.672823)
Supplement: Supplementary file 1 [file Data_Sheet_1.docx]

Supplementary Material


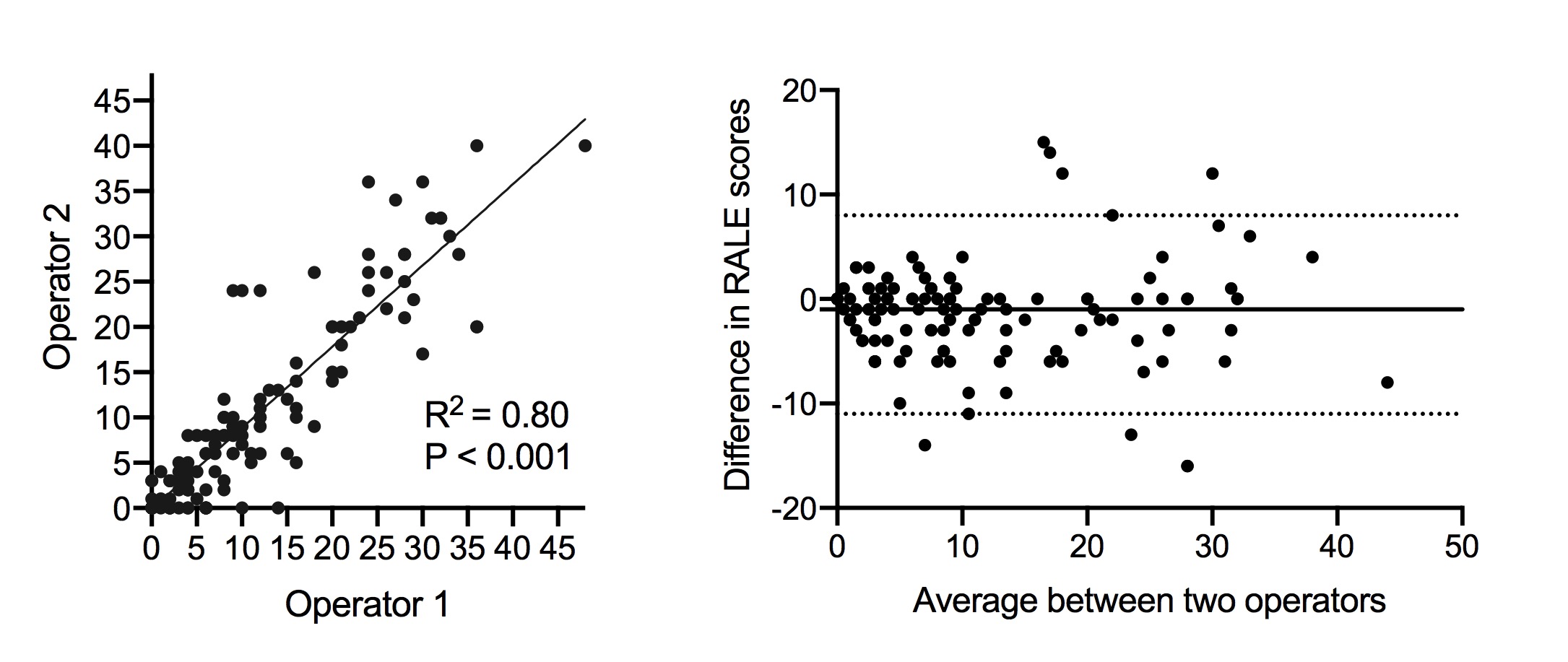


**eFigure 1:** Scatter plot and Bland–Altman plot showing agreement between two independent scorers for the Radiographic Assessment of Lung Edema (RALE) scores on 131 patients. Intraclass correlation coefficient (two-way, consistency type, average measures) among scorers was 0.97 (95% CI 0.94 – 0.98).


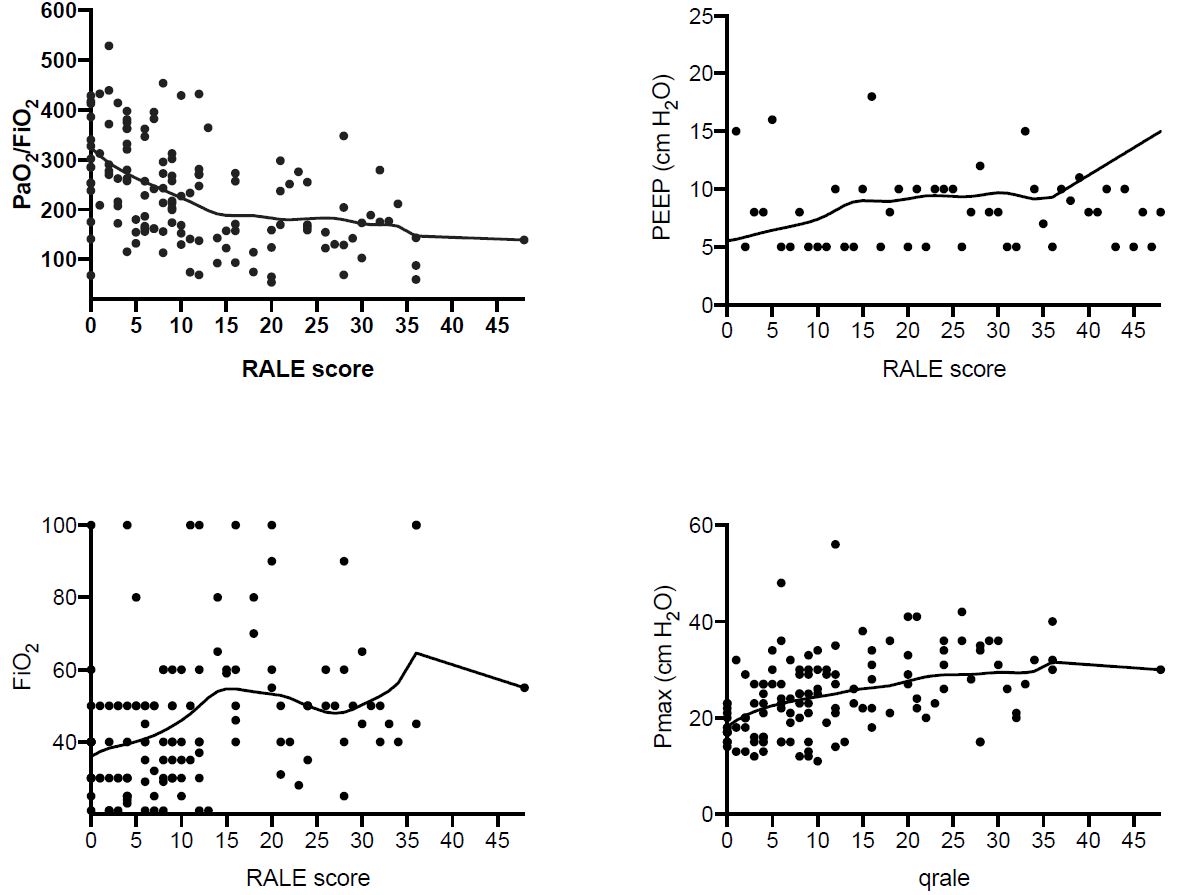


**eFigure 2.** Scatterplots assessing the relationship between the RALE score and PaO_2_/FiO_2_, positive end-expiratory pressure (PEEP), FiO_2_ and maximum airway pressure (Pmax). Locally weighted scatterplot smoothing (LOWESS) lines were fitted to allow trend visualization.


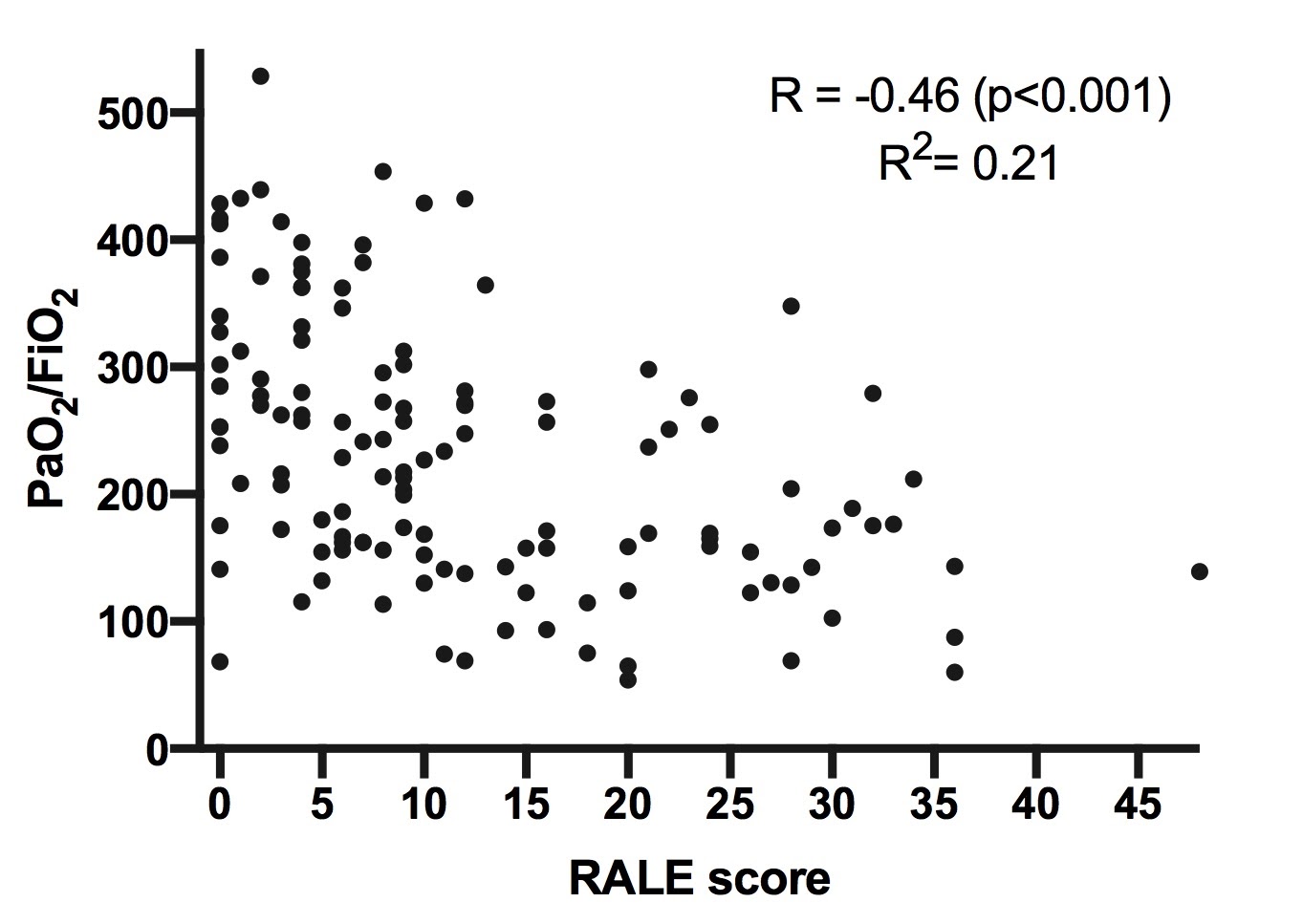


**eFigure 3.** Scatterplot assessing the relationship between the RALE score and the PaO2/FiO2 score in both ARDS and non ARDS patients.
